# Corticosteroid Pulse Therapy for Graves' Ophthalmopathy Reduces the Relapse Rate of Graves' Hyperthyroidism

**DOI:** 10.3389/fendo.2020.00367

**Published:** 2020-06-11

**Authors:** Rosario Le Moli, Pasqualino Malandrino, Marco Russo, Fabrizio Lo Giudice, Francesco Frasca, Antonino Belfiore, Riccardo Vigneri

**Affiliations:** ^1^Endocrinology, Department of Clinical and Experimental Medicine, Garibaldi-Nesima Medical Center, University of Catania, Catania, Italy; ^2^Institute of Crystallography, Structural Chemistry and Biosystems, CNR-ICCSB, Catania, Italy

**Keywords:** Graves' hyperthyroidism, corticosteroid pulse therapy, Graves' ophthalmopathy, relapse of Graves' hyperthyroidism, anti-thyroid drugs

## Abstract

**Background:** A course of anti-thyroid drugs (ATD) is the most common first line treatment for Graves' hyperthyroidism. However, hyperthyroidism relapse is frequent (30–70%). Due to the autoimmune nature of Graves' disease, the immunosuppressive treatment used for active Graves' orbitopathy (GO) may reduce the relapses after ATD discontinuation.

**Objective:** To evaluate the recurrence rate in Graves' patients who, in addition to standard ATD, were treated or not treated with parenteral methylprednisolone (MPDS) for GO.

**Methods:** Single-center retrospective study in a continuous series of 162 newly diagnosed Graves' patients, with or without GO, all gone into remission and followed-up until hyperthyroidism recurrence or at least 4 years after ATD discontinuation. Patients with moderate-severe active GO underwent middle dose MPDS treatment according to the EuGoGo guidelines. Cox proportional-hazard model was used to comparatively evaluate the risk of recurrence and the predictive factors in patients treated or not treated with MPDS pulse therapy.

**Results:** MPDS treatment was the most significant factor that independently correlated with a reduced risk of hyperthyroidism relapse (HR = 0.53, 95% C.I. = 0.31–0.89). FT3 and female sex were also independent protective factors, while age almost reached the significance level, *p* = 0.062. The efficacy of MPDS was very high in patients aged <40 years (42.1% decrease in relapses, *p* < 0.01) but it was not significant in older patients.

**Discussion:** Our study found that after ATD discontinuation the frequency of Graves' hyperthyroidism relapse was reduced in patients treated with MPDS pulse therapy for GO. This effect was more marked in young patients.

## Introduction

Graves' disease (GD) is an autoimmune disorder characterized by the excess production of thyroid hormones due to the overstimulation of the thyroid gland by thyrotropin (TSH)-receptor autoantibodies (TRAbs). GD is the most frequent cause of hyperthyroidism, with a prevalence of ~0.5% in the general population, with a female-to-male ratio of ~3:1. (1–3). The treatment options for GD are antithyroid drugs (ATD), radioiodine administration (RAI) or thyroidectomy (Tx). The most frequent long-term unfavorable consequences of these treatments are life-long hypothyroidism for Tx and RAI (4), radiation exposure for RAI (5) and hyperthyroidism recurrence for ATD (6, 7). Since a course of ATD (minimum 12–18 months) is now the most common first line treatment of GD, the risk of relapse is a relevant clinical problem. The overall relapse rate after a single course of ATD is ~50% although it may greatly vary from 30 to 70% in different studies, also depending from the length of the patient follow-up (7). Although several risk factors are believed to influence the relapse rate of GD after ATD treatment, the contribution of each risk factor is controversial and varies among studies (8–10). Recently, a predictive model based on simple clinical data detected at presentation of GD (patient age, goiter size, free thyroxine levels and TRAbs serum levels) and called GREAT (Graves' Recurrent Events After Therapy) has been proposed and validated (11–13).

Attempts to enhance the remission rate and to reduce hyperthyroidism recurrence after ATD treatment have been limited and have met little success. The autoimmune nature of GD and the curative effect of ATD are well established (14, 15).

Few studies have tested the effect of adding immunosuppressive agents to the standard ATD treatment on hyperthyroidism outcome. In this regard, a recent meta-analysis evaluated seven studies in which immunosuppressive drugs were added to the ATD treatment and concluded that the combined treatment reduced the relapse risk (16). However, the examined studies had a moderate/high risk of bias because of small effect and poor design (16).

More specifically, corticosteroids were used in four studies. In one study (17) the number of relapses was reduced in patients treated with the combined therapy; however, in the other study, methylprednisolone (MPDS) pulse therapy had only a temporary effect on the TBII decrease in Japanese patients, with no significant difference at 12 months after ATD discontinuation (18). The other two studies evaluated the effect of local thyroid treatment with corticosteroid and methimazole (MMI) in Chinese patients. In both series (19, 20) the relapse rate of hyperthyroidism was reduced.

Considering the clinical relevance of this issue, that the available data are mainly from patients of Asian ethnicity and the peculiar route of steroid administration, and considering also that high dose corticosteroid (pulse therapy) is the first line treatment for medium/severe active Graves' orbitopathy (GO), we retrospectively evaluated the effect of immunosuppressive corticosteroid therapy on the risk of hyperthyroidism recurrence rate in a continuous series of Graves' patients treated either with ATD alone or with corticosteroid pulse therapy for GO in addition to the standard ATD treatment.

## Methods

### Study Design

**Figure 1:**
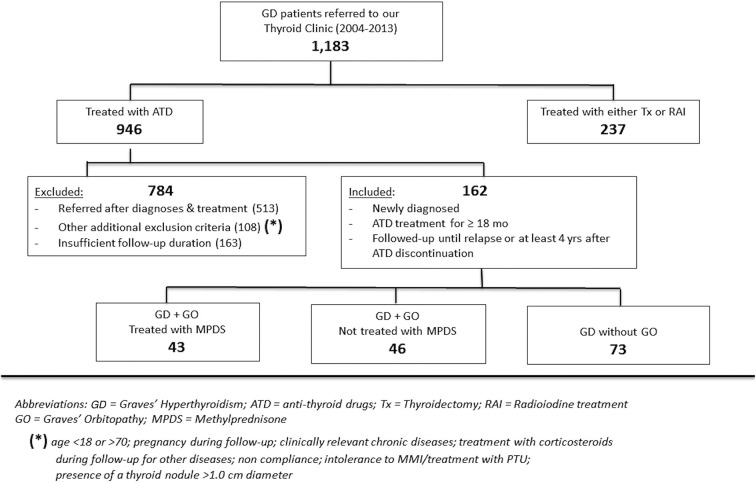
Flow chart of Graves' patients selection.

In this retrospective, observational, single-center study, 162 consecutive patients with newly diagnosed Graves' hyperthyroidism from 2004 to 2013 were selected from 1,162 Graves' hyperthyroidism patients referred to our thyroid tertiary center (Endocrinology, University of Catania, Sicily). The selection criteria are summarized in Figure 1. All 162 patients received ATD continuously for almost 18 months in most cases and underwent clinical and biochemical hyperthyroidism remission after ATD treatment (MMI in all cases). Additionally, as an inclusion criterion to enter the study, all patients were continuously followed-up in our Center until hyperthyroidism recurrence or until at least 4 years after ATD discontinuation.

Among the 162 selected patients, 43 (26.5%) who had moderate to severe active Graves' orbitopathy also received a course of treatment with parenteral MPDS.

### Graves' Hyperthyroidism

GD was diagnosed based on typical clinical signs and symptoms of hyperthyroidism confirmed by laboratory tests: TSH <0.4 mU/L, free thyroxine (FT4) > 23.0 pmol/L and/or free triiodothyronine (FT3) > 2.7 pmol/L and diffuse homogeneous uptake on thyroid scintigraphy (99mTc-pertechnetate).

### Anti-thyroid Treatment

As ATD treatment we employed a block and replace (B/R) regimen to better standardize the time intervals between one clinical examination and another (15–30 mg methimazole and L-thyroxine in a dose to maintain constantly normal thyroid function) in 122 cases while only titrated MMI administration was used in 40 patients in whom the combined regimen was not accepted. The treatment lasted a minimum period of 18 months or longer, until the normalization of clinical signs and laboratory data.

### Graves' Orbitopathy

GO severity and clinical activity stage were evaluated at presentation by trained investigators. GO severity was evaluated according to the EuGoGo guidelines (21). The lid fissure width was evaluated in millimeters by a router, and proptosis was evaluated with a Hertel exophthalmometer. Diplopia was classified according to the Gorman score. A complete ophthalmological evaluation was carried out by an expert ophthalmologist. The GO was defined as moderate to severe when eye disease had a sufficient impact on daily life with one or more of the following: lid retraction 2 mm or more, moderate or severe soft tissue involvement, exophthalmos three or more mm above 21, and inconstant or constant diplopia. GO was considered active when the clinical activity score (CAS) was ≥3 (22). Corticosteroid treatment for GO was administered to patients with moderate to severe active GO (21) and consisted of intravenous MPDS (Solumedrol; Pfizer, Karlsruhe, Germany) injections with a cumulative dose of 4.5 g subdivided in 12 weekly infusions (500 mg each week for 6 weeks followed by 250 mg week for 6 weeks); this treatment was initiated in all patients within 8 months after starting ATD and following the EuGoGo protocol (23, 24).

### Graves' Hyperthyroidism Remission

GD remission was defined as the condition of normal thyroid function after ATD discontinuation, documented both clinically and biochemically. Follow-up consisted of the control of clinical and laboratory parameters according to our well-established protocol which includes the first control visit 45 days after MMI discontinuation and then at 3, 6, and 12 months and every 6 months thereafter.

### Graves' Hyperthyroidism Relapse

GD relapse was defined as overt hyperthyroidism, occurring after documented remission lasting at last 3 months and confirmed by the clinical signs and symptoms of hyperthyroidism and by the laboratory values of serum TSH <0.4 mU/L in combination with elevated FT4 (>23.0 pmol/L) and/or elevated serum FT3 (>2.7 pmol/L) and diffuse homogeneous uptake on thyroid scintigraphy (99mTc-pertechnetate). In all the patients the following clinical parameters were recorded at baseline as they could potentially influence the risk of Graves' hyperthyroidism relapse: age, sex, BMI (body mass index), goiter size as assessed by ultrasound imaging (18), presence of GO and smoking behavior. Moreover, the GREAT class of each patient was also calculated as an overall indicator of the risk of hyperthyroidism recurrence after discontinuation of ATD treatment (11).

### Laboratory Measurements

Serum hormones were measured by microparticle enzyme immunoassay (Abbot AxSYM-MEIA) with inter-assay coefficients of variation of <10% over the analytical ranges of 1.7–46.0 pmol/L for FT3, 5.15–77.0 pmol/L for FT4 and 0.03–10.0 mU/L for TSH. The within-run and between-run precisions for FT3, FT4 and TSH assays showed coefficients of variation <5%. The TSH receptor antibodies (TRAbs) were measured by a radio-receptor assay (RADIM, Italy). Positivity was >9 UI/L.

### Statistical Analysis

The Mann Whitney U test was used to analyze the variables without normal distribution, and the chi-square test was used to study the categorical variables. Cut-off values for putative predictor variables were obtained by receiver operating characteristic (ROC) curve analysis. We carried out uni-and multivariate Cox regression analysis to identify the independent factors predictive of GD relapse and to determine the specific relevance of each factor. Independent predictors were identified by a *p*-value < 0.05. We included in the multivariate statistical model all variables that were significantly different by univariate analysis between the relapsed and not relapsed patients, and also smoke, FT4 and TRAb values that are known predictive factors for GD relapse. Additionally, to minimize the effects of selection bias from non-randomized treatment assignment, the potential outcome mean (POM), average treatment effect (ATE) and relative risk (RR) were calculated by combining the outcome and treatment models through an inverse probability weight regression adjustment (IPWRA) analysis.

The outcome was modeled as a function of sex, age at methylprednisolone administration, TRAbs positivity, cigarette smoke and FT3 values. The treatment was modeled as a function of sex, age at MPDS administration, B/R therapy and cigarette smoke. Thereafter, we assessed the standardized differences and considered a value <10% to be indicative of negligible imbalance in the baseline covariates between treated and untreated subjects (25). Statistical analyses were carried out using the STATA 15.1 statistical package (StataCorp LP, College Station, Texas, USA).

## Results

**Figure 2:**
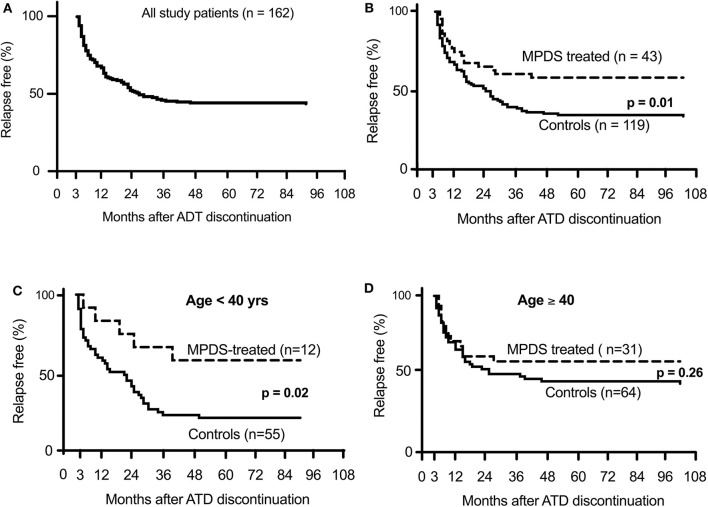
Percentage of relapse-free Graves' patients after discontinuation of anti-thyroid drug therapy. **(A)** shows all patients included in the study. **(B)** shown the difference between MPDS-treated and untreated patients. **(C,D)** show the different relapse rate according to patient age.

The clinical and biochemical characteristics of the 162 Graves' hyperthyroidism patients at presentation are shown in Table 1. The mean duration of ATD therapy was 26.4 ± 9.3 months. GO was present in 89/162 (54.9%) patients; however, according to the EuGoGo criteria, active GO of moderate to severe grade was diagnosed in only 43 patients who received early (within 4.1 ± 3.0 months from diagnosis) MPDS treatment. The most frequent side effect of this treatment was skin flushing after the i.v. MPDS (14 cases); of more relevance, hyperglycemia requiring 2–6 weeks treatment occurred in three patients (metformin was used in all cases), and development or worsening of hypertension occurred in six cases. After the ATD was withdrawn, the patients were followed-up for an average of 55.1 ± 27.1 months (range 19–124). Hyperthyroidism relapsed in 97/162 patients (59.9%) after a median disease-free interval of 24 months (range 3–103) after ADT discontinuation, while 65 patients (40.1%) remained euthyroid during the observation period (in no case shorter than 48 months after ADT withdrawal). The clinical and biochemical characteristics of the patients with or without Graves' hyperthyroidism relapse are shown in Table 1 and indicate that significant differences were observed for age (more frequent relapse in younger patients; *p* < 0.01) and for MPDS treatment (less frequent relapse in MPDS-treated patients, *p* < 0.005). The data indicate that the presence of GO was similar between relapsed and not relapsed patients (*p* = 0.5, Table 1). To further confirm this observation, we compared the 46 patients that had minimal or non-active GO and who were, therefore, not treated with MPDS (*n* = 46) with patients without GO (*n* = 73). No significant difference was observed between the two groups in terms of the clinical and biochemical parameters, except that the B/R scheme of ATD treatment was more frequent in the patients having GO who also had a shorter ATD treatment period (24.2 ± 8.2 vs. 28.6 ± 10.5 months). Additionally, the GREAT classes did not differ between these two groups (*p* = 0.14), indicating a similar risk of relapse at presentation. Therefore, these 119 patients were considered to be a single group (control group) to be compared with the 43 patients that had both Graves' hyperthyroidism and moderate to severe active GO and who received MPDS pulse therapy. When the control and the MPDS-treated groups were compared, most of the clinical and all the biochemical parameters at diagnosis were similar. Significant differences were observed only for age (higher in the group treated with MPDS, *p* = 0.011) and BMI (higher in the MPDS group). These patients were also more frequently treated with the B/R scheme (Table 2). The GREAT classes were not significantly different (*p* = 0.38) between the groups, confirming that the risk of hyperthyroidism relapse evaluated at presentation was similar. Remarkably, the prevalence of hyperthyroidism relapse after ATD withdrawn was significantly lower in the patients who received MPDS (*p* = 0.005). In addition, in these patients the disease-free interval was significantly longer (*p* = 0.014) than that in the control group. Hazard ratios indicated that MPDS treatment, sex and FT3 were the only significant factors predicting the risk of relapse, while age almost reached the significance level (*p* = 0.062) (Table 3). The role of MPDS therapy in reducing the risk of relapse was further confirmed by the IPWRA method. Indeed, we observed that the POM (i.e., the percentage of relapse) was 65.5% (95% CI = 56.8–74.3%) and 40.5% (95% CI = 27.3–53.6%) for patients who were untreated or treated with MPDs, respectively. Thus, if no patient was treated with MPDS the risk of relapse would have been 1.62 times higher (95% CI = 1.15–2.29, *p* = 0.006) than if all the patients had received the MPDS treatment. As shown in Table 4 and Figure 2 the beneficial effect of pulse MPDS treatment on reducing relapse was more marked in young patients (<40 years), effectively counteracting the higher propensity toward relapse among patients in this age group (10). In contrast, MPDS pulse therapy did not significantly reduce the risk of relapse in patients older than 40 years (*p* = 0.084) (Table 4 and Figure 2).

**Table 1:** Clinical and laboratory characteristics of the studied patients at presentation subdivided according to Graves' ophthalmopathy presence and therapy with methylprednisolone.

	**GD without GO**	**GD + GO without MPDS therapy**	**GD + GO + MPDS therapy**	***P***
Patients	73	46	43	
Age (years, m ± SD)	38.9 ± 13.6	41.4 ± 11.1	45.5 ± 11.1	0.021
Sex (m/f)	17/56	16/30	11/32	0.38
BMI (Kg/m^2^, m ± SD)	22.1 ± 2.6	23.5 ± 3.6	29.6 ± 5.0	<0.001
Smoking	25 (34.2)	17 (37.0)	22 (51.2)	0.18
TSH (μU/ml, m ± SD)	0.03 ± 0.11	0.01 ± 0.03	0.02 ± 0.03	0.35
FT3 (pmol/L, m ± SD)	13.8 (7.2)	14.6 (10.3)	12.5 (6.6)	0.45
FT4 (pmol/L, m ± SD)	47.7 (20.7)	42.5 (21.4)	41.8 (21.0)	0.24
TRAB (IU/L, median, IQR)	37.1 (13.5–80.3)	27.3 (6.3–87.3)	25.4 (12.6–65.9)	0.55
Thyroid volume (ml, m ± SD)	21.8 ± 10.9	19.4 ± 11.4	22.2 ± 14.0	0.46
**GREAT SCORE**				
Class 0	18 (24.7%)	18 (39.1%)	16 (37.2%)	0.20
Class 1	39 (53.4%)	23 (50.0%)	23 (53.5%)	
Class 2	16 (21.9%)	5 (10.9%)	4 (9.3%)	
ATD duration (months, m ± SD)	28.6 ± 10.5	24.2 ± 8.2	25.1 ± 7.5	0.024
B/R therapy	41 (56.2%)	42 (91.3%)	39 (90.7%)	<0.001
GD relapse	51 (69.9%)	28 (60.9%)	18 (41.9%)	0.012
Disease free interval (months, m ± SD)	26.5 ± 25.7	24.7 ± 20.6	37.0 ± 28.9	0.045

*GD, Graves' Disease; GO, Graves' Ophthalmopathy; MPDS, methylprednisolone*.

**Table 2:** Clinical and laboratory characteristics of untreated and MPDS- treated Graves' disease patients.

	**MPDS untreated**	**MPDS treated**	***p***
N. cases	119	43	
Female gender	86 (72.3%)	32 (74.4%)	0.79
Age (years, m ± SD)	39.8 ±12.7	45.5 ± 11.1	0.011
BMI (Kg/m^2^, m ± SD)	23.0 ± 3.3	29.6 ± 5.0	<0.001
Smoking	42 (35.3%)	22 (51.2%)	0.06
TSH (μU/ml, m ± SD)	0.03 ± 0.09	0.02 ± 0.03	0.65
FT3 (pmol/L, m ± SD)	14.1 ± 8.5	12.5 ± 6.6	0.25
FT4 (pmol/L, m ± SD)	45.7 ± 21	41.8 ± 21	0.3
TRAb (IU/L, median, IQR)	25.4 (12.6–65.9)	32.9 (12.6–80.3)	0.64
Thyroid volume (ml, m ± SD)	20.9 ± 11.1	22.2 ± 14	0.54
**GREAT SCORE**			
Class 0	36 (30.3%)	16 (37.2%)	0.38
Class 1	62 (52.1%)	23 (53.5%)	
Class 2	21 (17.6%)	4 (9.3%)	
ATD duration (months, m ± SD)	26.9 ± 9.9	25.1 ± 7.5	0.28
B/R treatment	83 (69.7%)	39 (90.7%)	0.006
Follow-up after ATD discontinuation (months, m ± SD)	52.7 ± 26.2	62 ± 28.5	0.051
GD relapse	79 (66.4%)	18 (41.9%)	0.005
Disease free interval (months, m ± SD)	25.8 ± 23.8	37 ± 28.9	0.014

**Table 3:** Variables associated with the Graves' disease relapse in the study population (uni- and multivariate Cox regression analysis).

	**Univariate analysis**		**Multivariate analysis**	
	**Hazard ratio (95% CI)**	***P***	**Hazard ratio (95% CI)**	***P***
Age <40 yrs	1.57 (1.05–2.34)	0.029	1.48 (0.98–2.22)	0.062
Male sex	1.58 (1.02–2.43)	0.038	1.56 (1.00–2.43)	0.048
BMI	0.95 (0.87–1.03)	0.18		
Smoking	1.23 (0.82–1.85)	0.31	1.27 (0.84–1.93)	0.26
GO	0.67 (0.45–1.00)	0.048		
FT3	1.03 (1.00–1.06)	0.02	1.04 (1.00–1.07)	0.036
FT4	1.00 (0.99–1.01)	0.63	0.99 (0.98–1.01)	0.24
TRAb	1.00 (0.99–1.00)	0.22	1.00 (0.99–1.00)	0.27
Thyroid volume	1.00 (0.99–1.02)	0.58		
**GREAT SCORE**				
1	1.04 (0.67–1.63)	0.86		
2	1.40 (0.76–2.56)	0.28		
ATD duration	1.12 (0.75–1.67)	0.58		
B/R pulse therapy	1.14 (0.71–1.84)	0.58		
MPDS therapy	0.52 (0.31–0.88)	0.014	0.53 (0.31–0.89)	0.017

*BMI, body mass index; GO, Graves' ophthalmopathy; TRAb, thyrotropin receptor antibody; ATD, anti thyroid drug; B/R, block and replace; MPDS, methylprednisolone*.

**Table 4:** Inverse probability weight regression analysis (IPWRA) for Graves' disease relapse according to MPDS pulse therapy and patient age.

	**All patients (*****n*** **=** **162)**		**Age** **<** **40 y (*****n*** **=** **67)**		**Age** **≥** **40 y (*****n*** **=** **95)**	
	**MPDS –**	**MPDS +**	**MPDS –**	**MPDS +**	**MPDS –**	**MPDS +**
POM	65.5% (56.8–74.3)	40.5% (27.3–53.6)	78.9% (68.1–89.6)	53.5% (36.5–70.5)	56.4% (44.1–68.7)	38.0% (22.9–53.1)
ATE	−25.1% (−40.6 to −9.6)		−25.4% (−45.0 to −5.8)		−18.4% (−37.6 to 0.8)	
RR	1.62^*^ (1.15–2.29)		1.47^*^ (1.05–2.07)		1.48^ns^ (0.95–2.33)	

*POM, potential outcome mean (i.e., the percentage of Graves' disease relapse); ATE, average treatment effect (i.e., the difference of relapse between MPDS-treated and untreated patients); RR, relative risk (i.e., the risk ratio of MPDS-treated to untreated patients)*.

*Values between brackets indicate 95% confidential intervals*.

* *P < 0.05*.

*ns, not significant*.

## Discussion

Hyperthyroidism due to Graves' disease is a common disorder that is currently treated with ATD in most cases. However, recurrence of hyperthyroidism after ATD discontinuation is frequent, with cumulative occurrence being time-dependent even if most cases relapse within 2 years after therapy withdrawal. Due to the autoimmune nature of the disease, immunosuppressive drugs in combination with ATD treatment seem to be a rationale treatment from an etiopathogenetic approach. This approach, however, has not been adequately studied because of the potentially severe adverse events associated with chronic immunosuppression. Corticosteroids, which represent the standard treatment for many autoimmune diseases, can cause serious side effects, including metabolic disturbances, osteoporosis and hypertension. In this study we observed no major side effect in the patients treated with MPDS pulse therapy, although severe adverse events have been reported (26, 27). However, the risks inherent to chronic corticosteroid administration are acceptable for treating severe orbitopathy, which is a frequent complication of Graves' disease. MPDS pulse therapy is, in fact, the first line treatment for patients with moderate to severe active GO.

The present study, although with limitations, shows that immunosuppression with MPDS for active GO significantly reduces the risk of hyperthyroidism relapse. This effect was already nearly significant at 1 year after MMI withdrawal (*p* = 0.07), and further increased up to 4 years, when it reached a plateau with a >42% decrease in the relapse rate relative to patients not treated with MPDS (*p* = 0.01) (Figure 2). Although late recurrence may occur, the prognosis is excellent after 4 years without relapse (28). Moreover, even in cases of relapse, MPDS-treated patients benefit from a longer disease-free interval (*p* = 0.014) (Table 2).

The multivariate analysis indicated that MPDS pulse therapy most effectively predicts reduced risk of GD recurrence and is a more effective predictor than patient sex, age or FT3 which are well-known risk factors for relapse (Table 3) (10, 12, 29).

In our series, other risk factors for GD relapse such as high TRAbs concentrations, large goiter size were not significantly different between relapsed and not relapsed patients and were not included in multivariate analysis. In contrast to other studies (30) we found no association between GO and an increased risk of hyperthyroidism recurrence. In any case, we found that in young GO patients, that in some studies are considered at higher risk of recurrent hyperthyroidism, MPDS treatment reduced the relapse rate to levels lower than those in patients without GO.

A remarkable observation of our study is that MPDS pulse treatment showed different effects at different patient ages. It is well known that the remission rate is lower and the relapse rate is higher in young age patients with Graves' disease (31). The Cox regression and the IPWRA analyses (Table 4) as well as the Kaplan-Meier curves (Figure 2) indicated that MPDS treatment was highly effective in reducing the relapse rate in younger patients (<40 years) but not in older Graves' disease patients. The reason for the different age-related responses to corticosteroids is unclear, as is the reason why disease outcome tends to be worse in younger patients. Higher levels of TRAbs and circulating thyroid hormones and larger thyroid volume in younger patients have been previously discussed as possible causative factors for worse Graves' disease outcome among younger patients (29). We did not observe similar differences in our series (TRAbs difference *p* = 0.99; FT4 *p* = 0.93 and thyroid volume *p* = 0.81 between patients younger or older than 40 years). Corticosteroid absorption, distribution and efficacy on immunocompetent cells are possible reasons for greater efficacy in younger patients.

These potential differences include the effects of glucocorticoid receptor activation on a series of regulators. Both non-genomic effects such as cytokine and growth factors cross-talk (32–34) and epigenetic modifications in many target tissues may determine a different individual responses across patient's lifespan (35). There is no evidence that a longer treatment with ATD is associated with a reduced relapse rate (24), but when combined with steroids ATD treatment may contribute to a higher remission rate. However, in our study ATD treatment length was no longer in patients treated with MPDS.

From a clinical point of view, among young patients for whom surgery or radioiodine is usually indicated due to the patient's higher likelihood of failure to respond to ATD treatment, MPDS pulse therapy, required for the presence of GO, may reverse the therapeutic advice in favor of ATD.

Our study has some limitations inherent to retrospective studies and lacks familial and genetic characteristics of the studied patients (11, 16, 30).

In addition, repeated TRAbs analysis during follow-up might have provided useful information. On the other hand the long 4-year follow-up during remission, the standardized protocol with complete data from all patients and the robust statistical analyses of the results are strengths of this study.

## Conclusions

In conclusion we observed that Graves' patients treated with MPDS pulse therapy for GO have a significantly reduced risk of hyperthyroidism relapse, confirming the important role of immunosuppression in the clinical outcomes of Graves' patients. This steroid effect is more relevant in younger patients who more frequently undergo hyperthyroidism relapse. When GO is present, in these patients the combined medical treatment reduces the relapse risk and should be considered as an alternative to more definitive treatments such as surgery or radioiodine. However, a prospective study is necessary to confirm these statements.

## Data Availability Statement

All relevant data are contained within the article and immediately available. Request to access the dataset should be directed to corresponding authors or to segmeint@unict.it.

## Ethics Statement

The studies involving human participants were reviewed and approved by Ethics Committee Garibaldi Nesima Hospital - Catania. Written informed consent for participation was not required for this study in accordance with the national legislation and the institutional requirements.

## Author Contributions

RL and RV: conception and design of the study. RL, PM, MR, and FL: data collection. RL and PM: statistical analysis. RL, FL, PM, and MR: figures. RL, RV, AB, FF, PM, and MR: writing and revision of the manuscript. All authors read and approved the final manuscript.

## Conflict of Interest

The authors declare that the research was conducted in the absence of any commercial or financial relationships that could be construed as a potential conflict of interest.

## Abbreviations

GH: Graves&#8217
hyperthyroidism
GO: Graves&#8217
ophthalmopahty
ATD: anti thyroid drugs
B/R, block and replace therapy, MPDS: metilprednisolone
EuGoGo: The European Group on Graves&#8217
Orbitopathy
RAI: radioiodine administration
Tx: thyroidectomy
GREAT: Graves&#8217
Recurrent Events After Therapy
FT3: triiodothyronine
FT: thyroxine or tetraiodothyronine
TSH: thyroid stimulating hormone
TRAB: thyrotropin (TSH)-receptor autoantibodies
TBII: TSH binding inhibitor immunoglobulins
MMI: methimazole
ROC: receiver operator characteristics
POM: potential outcome mean
ATE: average treatment effect
RR: relative risk
IPWRA: inverse probability weight regression adjustment analysis.
